# Exploration of the adsorption capability by doping Pb@ZnFe_2_O_4_ nanocomposites (NCs) for decontamination of dye from textile wastewater

**DOI:** 10.1016/j.heliyon.2019.e02412

**Published:** 2019-09-19

**Authors:** Ganesh Jethave, Umesh Fegade, Sanjay Attarde, Sopan Ingle, Mehrorang Ghaedi, Mohammad Mehdi Sabzehmeidani

**Affiliations:** a School of Environmental and Earth Sciences, KBC North Maharashtra University, Jalgaon, MS, India; b Bhusawal Arts, Science and P. O. Nahata Commerce College, Bhusawal, MS, India; c Chemistry Department, Yasouj University, Yasouj, Iran; d Chemical Engineering Department, Yasouj University, Yasouj, Iran

**Keywords:** Environmental science, Adsorption mechanism, Isotherms, Pb@ZnFe_2_O_4_, Congo red dye, Statistical model

## Abstract

In the present research article we explore the synthesis method and adsorption capability of ZnFe oxides nanocomposites by using Pb as dopant. A conventional and simple batch adsorption method is selected and optimized. Pb@ZnFe_2_O_4_ NCs were fabricated by facile method i.e. co-precipitation method and characterized by FESEM, XRD, IR, EDX. The removal of dye has monitored by UV method.

An outstanding result is obtained as adsorption efficiency of 1042 mg g^−1^ shows more significant performance than currently available bench-mark adsorbents. The optimized parameters pH 7.1, Adsorbent Mass: 50 mg, Initial Dye Concentration: 150 mg/l and Agitation Time: 90 min results in 96.49 % removal of CR (Congo red) dye. A CCD (central composite design) is applied to evaluate the role of adsorption variables. Based on its excellent performance, cost effectiveness, facile fabrication and large surface area, the Pb@ZnFe_2_O_4_ has considerable potential for the manufacture of cost effective and efficient adsorbents for environmental applications.

## 1 Introduction

Important concerns have been caused by colors and dyes in effluents for their harmful influence on ecosystems and can be carcinogenic to living organisms [1, 2, 3]. Among various methods for the treatment of effluents, adsorption is applicable approach owing to its advantages over other methods like its low cost [4, 5]. Thus making the process more environment friendly and economical, removal of synthetic dye pigments using several adsorbents have been reported in literature [6]. Various adsorbents including activated carbons, clays, zeolites, polymeric substances, etc., have been used for dye removal from effluents [7, 8]. Nowadays, adsorbents have a high preference in removing organic pollutants such as dyes, antibiotics and pesticides [9, 10]. It is notable that dyes are categorized as toxic, hazardous, carcinogenic and mutagenic material [11, 12]. So it becomes extremely fundamental to expel it from textile industrial waste water emanating before releasing it in to adjacent consumable water assets. Also, Congo red (CR) is one of the most used dye in the textile industry that is difficult to decompose due to its complicated aromatic structure [13].

Nanoparticle as an adsorbent is the most adaptable and revolutionary application among them. Metal oxides have huge amount of binding sites, which are responsible to hold the organic molecule of opposite charges due to fascinating forces [14, 15]. It has scientific proof that in the opposite charges there is a strong attraction among each other than any other forces [16, 17, 18]. Considering these characteristics quality of nanoparticles we explore it and used for decontamination of dyes thrown out as a waste water from textile industries in natural water sources. Metal Oxide nanoparticles have been efficiently used for the adsorption of dyes from solutions. Metal oxide nanoparticles have recently gained attraction due to their interesting textural properties viz. large surface area for the adsorption of dyes from wastewater [19]. Konicki et al. synthesized ZnFe_2_O_4_ spinel ferrite nano particles as the magnetic adsorbent used for the removal of Acid Red 88 from aqueous solution [20]. Zeng et al. prepared magnetically separable Ni_0.6_ Fe_2.4_ O_4_ for rapid adsorption of CR dye in aqueous solution [21]. We chose Zn, Fe transition metals due to their adsorption characteristic nature reported in previous literature [22, 23]. We prepare a defect in ZnFe_2_O_4_ nanocomposites (NC) by doping Lead (Pb) in very tiny amount so that it minimize its toxic effects and maximize adsorption capability of nanocomposites.

In the present work, Pb@ZnFe_2_O_4_ nanoparticles were successfully synthesized by co-precipitation method and used for removal of CR. The analysis such as by FESEM, XRD, IR, EDX has confirmed synthesis of Pb@ZnFe_2_O_4_ nanoparticles. The effect of contact time, temperature, concentration of CR and solution pH on the adsorption behavior of CR were investigated. Based on the test of the isotherm, kinetic and thermodynamic data, an adsorption mechanism has been proposed. In addition, the treatment a real wastewater system containing CR, has been further checked.

## 2 Experimental

### 2.1 Preparation of Pb@ZnFe_2_O_4_

**Fig. 1:**
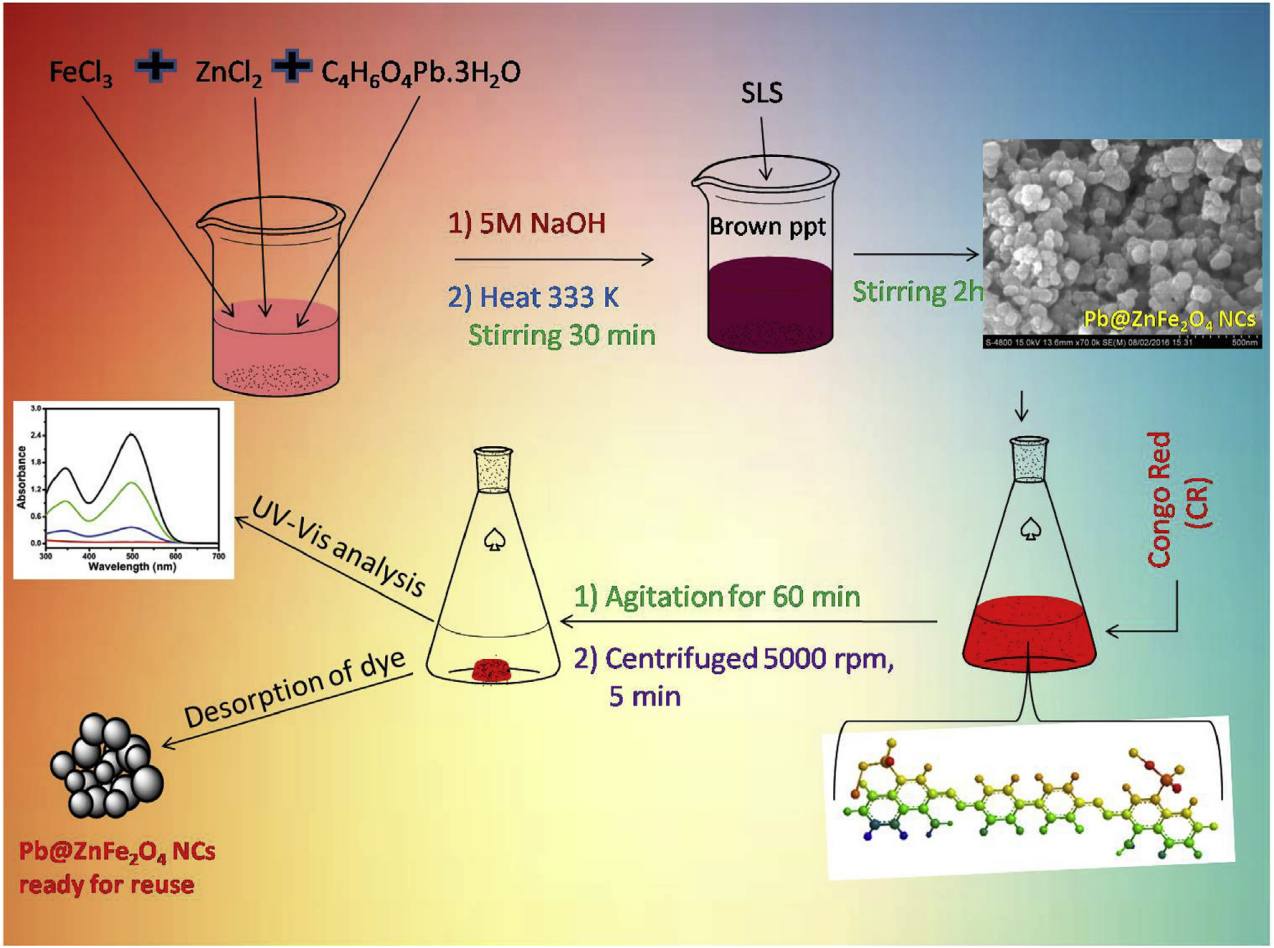
Schematic representation of synthesis of Pb@ZnFe_2_O_4_ NCs and Adsorption prossess of CR.

The Pb@ZnFe_2_O_4_ was synthesized by an easy and savvy co-precipitation method after magnetic stirring. Firstly 2 moles of FeCl_3_ and 1 moles of ZnCl_2_ were added to 200 ml DW and minute amount of lead acetate was added for doping purpose, similarly sodium lauryl sulphate (SLS) added as a surfactant solution which controls the size of trimetallic oxide nanoparticles. The solution was stirred for 5 min at 60 °C. Then 50 ml NaOH of 5 M concentration solution was slowly mixed into above solution under vigorous stirring, which was stirred at 60 °C for another 2 h, leading to smaller and more homogenized particles (Fig. 1). Until the suspension was reduced to pH 7.5, a substance produced by it was washed gently with DW through a magnetic decantation and brown suspension is formed. The suspension was separated by means of suction pump and then dried in vacuum at 100 °C for 12 h. To obtain Pb@ZnFe_2_O_4_ nanoparticles, prepared nanomaterial calcinations done for 2 h at 300 °C in an oven. At long last, the darker Pb@ZnFe_2_O_4_ powder was acquired, which was gathered for further examination.

### 2.2 Batch adsorption studies for Congo red

Adsorption experiments using Pb@ZnFe_2_O_4_ was done to assess the decontamination of CR dye from liquid phase. The adsorption tests were performed by set (batch) process. 50.0 mg of Pb@ZnFe_2_O_4_ was taken in a progression of 100.0 ml conical flasks holding 50.0 ml of CR dye solutions whose concentrations ranged from 10.0-200.0 mgL^-1^. The Conical flasks were shaken for a settled time interim (5 min–120 min at RT). The agitation time of 60 min was fixed during kinetic experiments. The pH of dye solution varied from 5.1-9.1 for the pH effect study on adsorption. The solution allowed to settle for a couple of minutes and centrifuged for 5 min at 5000 rpm. Subsequently 1–10 ml an intercourse of supernatant has been examined by means of a UV-Visible Spectrophotometer (UV-1800). Absorbance was recorded at 498 nm wavelength. Efficiency of removal of dye using Pb@ZnFe_2_O_4_ was assessed applying following equations.

(1) $${\text{q}}_{e}=\frac{\left(C_{0}-C_{e}\right)V}{m}$$

(2) $${\text{R}}_{\text{Percentage ​removal}}\left(\%\right)=\frac{\left(C_{0}-C_{t}\right)}{C_{0}}\times 100$$

Where: q_e_ = quantity of CR dye taken up by adsorbents, mgL^−1^; C_0_ = initial concentration of the CR dye (mg L^−1^); C_e_ = dye concentration after adsorption, mg L^−1^; C_t_ = dye concentration after adsorption time, mg L^−1^; V - Volume of the CR dye, L and m = Mass of the adsorbent, g.

## 3 Results and discussion

### 3.1 Structure and size of the Pb@ZnFe_2_O_4_

**Fig. 2:**
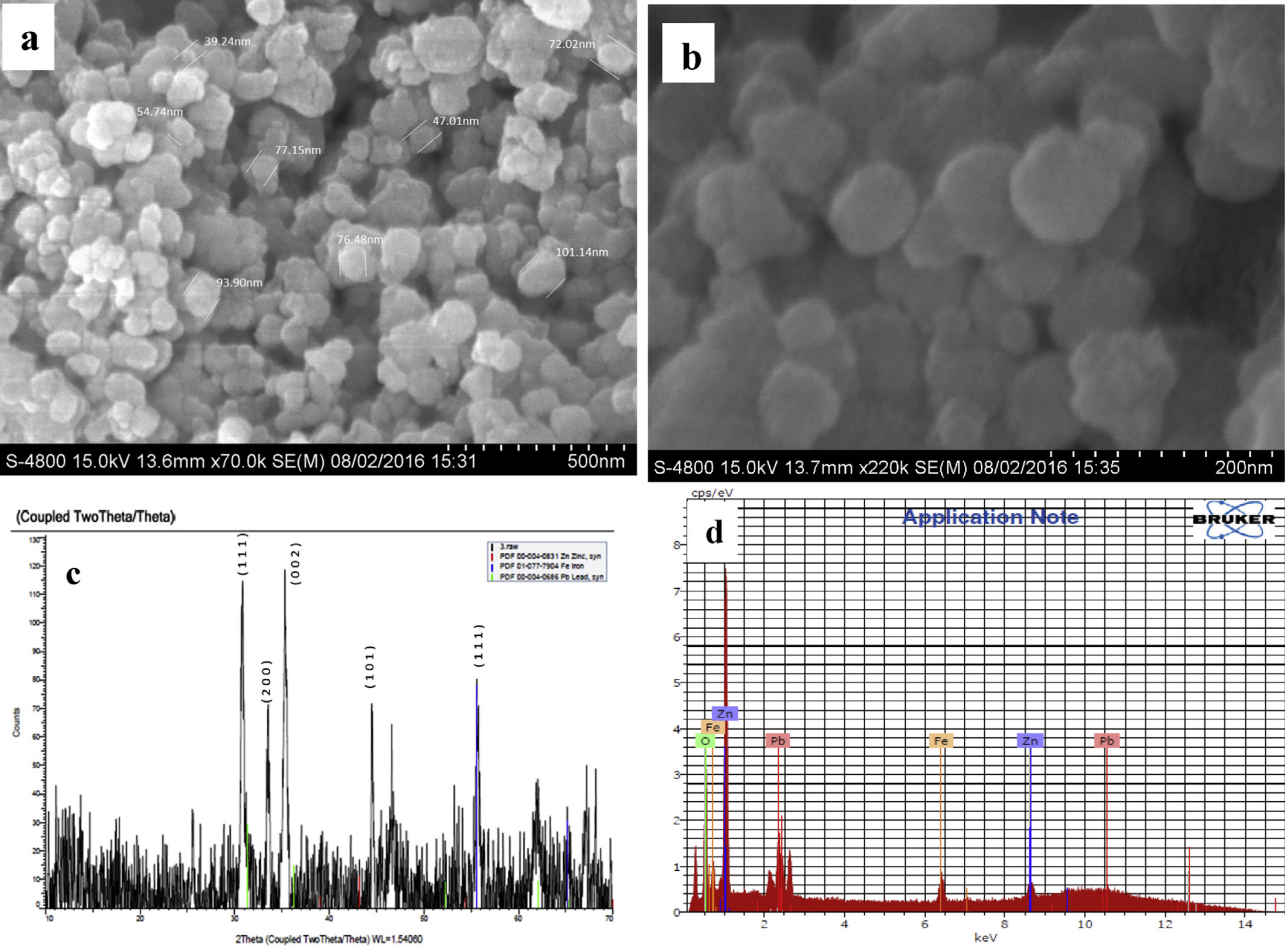
(a) SEM Images of Pb@ZnFe_2_O_4_ average particle size 70.21 nm at low magnification, (b) high magnification for the closed image of nanoparticles, (c) X-ray diffraction pattern of Pb@ZnFe_2_O_4_, (d) EDX spectrum of the as-prepared Pb@ZnFe_2_O_4._

A typical morphological image of the developed Pb@ZnFe_2_O_4_ has been shown by Fig. 2a and b. In Fig. 2a it is plainly seen that the Pb@ZnFe_2_O_4_ is suspended of a large quantity of consistent particles size about 70.21 nm. The high-amplification SEM picture in Fig. 2b uncovers that the surfaces of the Pb@ZnFe_2_O_4_ are fairly harsh. Crystal structures of the Pb@ZnFe_2_O_4_ sample investigated by Powdered X-ray Diffraction. To study crystalline nature of prepared Pb@ZnFe_2_O_4_ sample, the PXRD patterns recorded in the 2θ range 20°–90°. Fig. 2c depicts the XRD pattern of the as-prepared Pb@ZnFe_2_O_4_. All the diffracted peaks of Pb@ZnFe_2_O_4_ are labeled. Six diffraction peaks were observed at 2θ = 31.306°, 36.267°, 36.297°, 38.993°, 43.233° and 55.598°corresponding to (1 1 1), (2 0 0), (0 0 2), (1 0 0), (101) and (1 1 1) crystal planes of cubic Pb@ZnFe_2_O_4_, respectively. This exposed that the Pb@ZnFe_2_O_4_ has polycrystalline structure. The EDX analysis also shows that the atomic ratio of zinc, iron and lead to oxygen is close to 0.5:0.15:0.01:1 (Fig. 2d.).

Supplementary Fig. S2 a and b shows the IR spectra of Pb@ZnFe_2_O_4_ and CR adsorbed- Pb@ZnFe_2_O_4_ in the range 600–4000 cm^−1^ respectively. The possible interaction between CR and the Pb@ZnFe_2_O_4_ was illustrated by FT-IR bands of CR/Pb@ZnFe_2_O_4_. The characteristic bands at 1620, 1224 and 1176 cm^−1^ belongs to asymmetric S=O stretching, symmetric stretching of S=O and aromatic C=C vibration in CR molecule, respectively, disappeared in supplementary Fig. 2b, due to the formation of strong hydrogen bonds between SO^−3^ groups in the CR molecule and hydroxyl groups of Pb@ZnFe_2_O_4_ at acidic pH.

### 3.2 pH effect study on adsorption of CR dye

Role of pH is very important in adsorption mechanism because adsorption capacity is greatly influenced by adsorption system pH. Different adsorbates possess different most favorable pH values for adsorption. In this study, Fig. 6 shows that the original pH of adsorbate was varied from pH 5.0 to 9.0. The highest adsorption capability was observed between pH 5.5–6.5 for CR onto Pb@ZnFe_2_O_4_. The % adsorption increases by growing pH and reach maximum near pH 6 and then decreased at higher pHs. The value of pH_ZPC_ for Pb@ZnFe_2_O_4_ adsorbent is 7.25. The zero point of charge is a central portrayal of a mineral surface where the aggregate convergence of surface anionic destinations is equivalent to the aggregate concentration of outside cationic site. At pH > zpc the exterior has a net negative or anionic charge and the surface would contribute in cation fascination and cation trade responses. At pH < ZPC, The surface has total positive or cationic charge, and the surface contributes to attracting ions, and participates in ion exchange reactions [24, 25, 26].

### 3.3 Mechanism of the adsorption in terms of change in pH

Metal oxides surfaces are generally sheltered by hydroxyl groups that differ in forms at different pH. Since pH of CR solution increases, the adsorption proportion decreases due to deprotonation i.e. succeeding deprotonation of hydroxyl group happens on the adsorbent exterior and due to this deprotonation electrostatic repulsion between negatively charged sites of adsorbent and dye anions occur. In addition to this, there was also antagonism between OH^−^ (at elevated pH) and dye anions to occupy positively charged adsorption sites. Also, by coordination consequence between amine groups and metal ions at the extreme site of CR molecules, CR can get adsorbed on the metal oxide exterior. Subsequently it is noticeable that metal oxides with the higher surface region and below pHzpc can adsorb more anionic CR molecules [27].

(3) $$-\text{Zn}-\text{Fe}-\text{Pb}-\text{O}-\text{H }+{\text{ H}}^{+}⇌-\text{Zn}-\text{Fe}-\text{Pb}-\text{O}-{\text{H}}_{2}^{+}$$

**Fig. 3:**
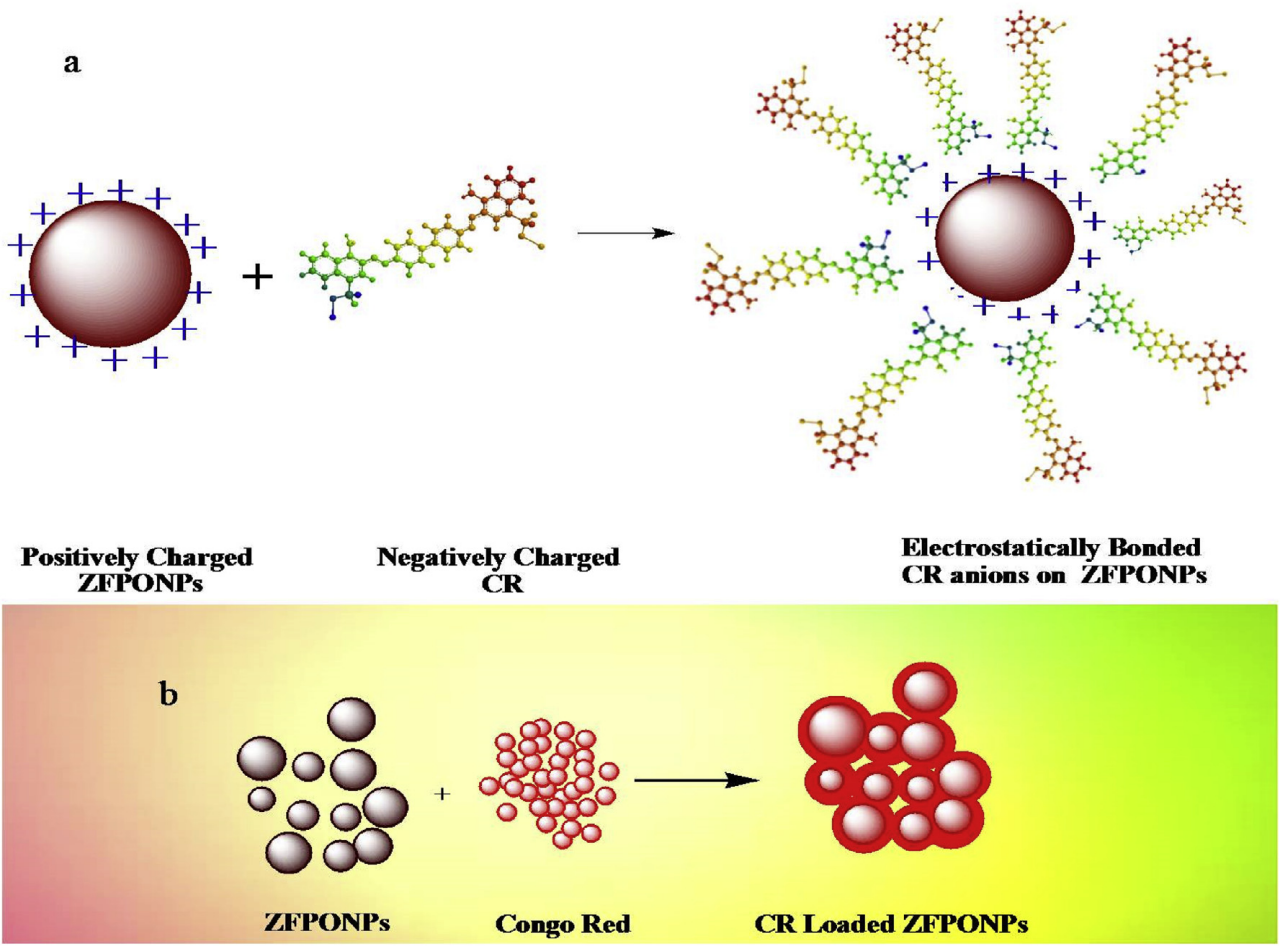
a) electrostatic attraction of charged particle of dye molecule to oppositely charged surface of Metal nanoparticles, b) Schematic picture of the adsorption of CR dye on the surface of Pb@ZnFe_2_O_4_ Nanocomposite.

In contrast, the predominant charges on the Pb@ZnFe_2_O_4_ at acidic pH are positive and because of anionic dye species, it seems that the fundamental mechanism of the adsorption is electrostatic attraction. Resting on pH 5.9 and below, significant high electrostatic attractions exist between the oppositely charged surface of the Pb@ZnFe_2_O_4_ (positive) and CR dye (negative). The negative charge on Pb@ZnFe_2_O_4_ increases with increasing pH of the system. With the expanding pH esteems, the adsorption of Congo Red on Pb@ZnFe_2_O_4_ tends to diminish, which can be clarified by the expanding electrostatic repulsion between the anionic dye species and adversely charged Pb@ZnFe_2_O_4_ surfaces. Also lesser adsorption at basic pH was observed as a result of presence of surplus OH^−^ ions in the solution destabilizing anionic dye and contending with the dye anions to get adsorbed on binding sites. Low pH leads to a boost in H^+^ ion concentration in the system and the surface of Pb@ZnFe_2_O_4_ acquires positive charge by absorbing H^+^ ions and consequently additional anionic dye amount of adsorption is observed (Fig. 3).

**Fig. 4:**
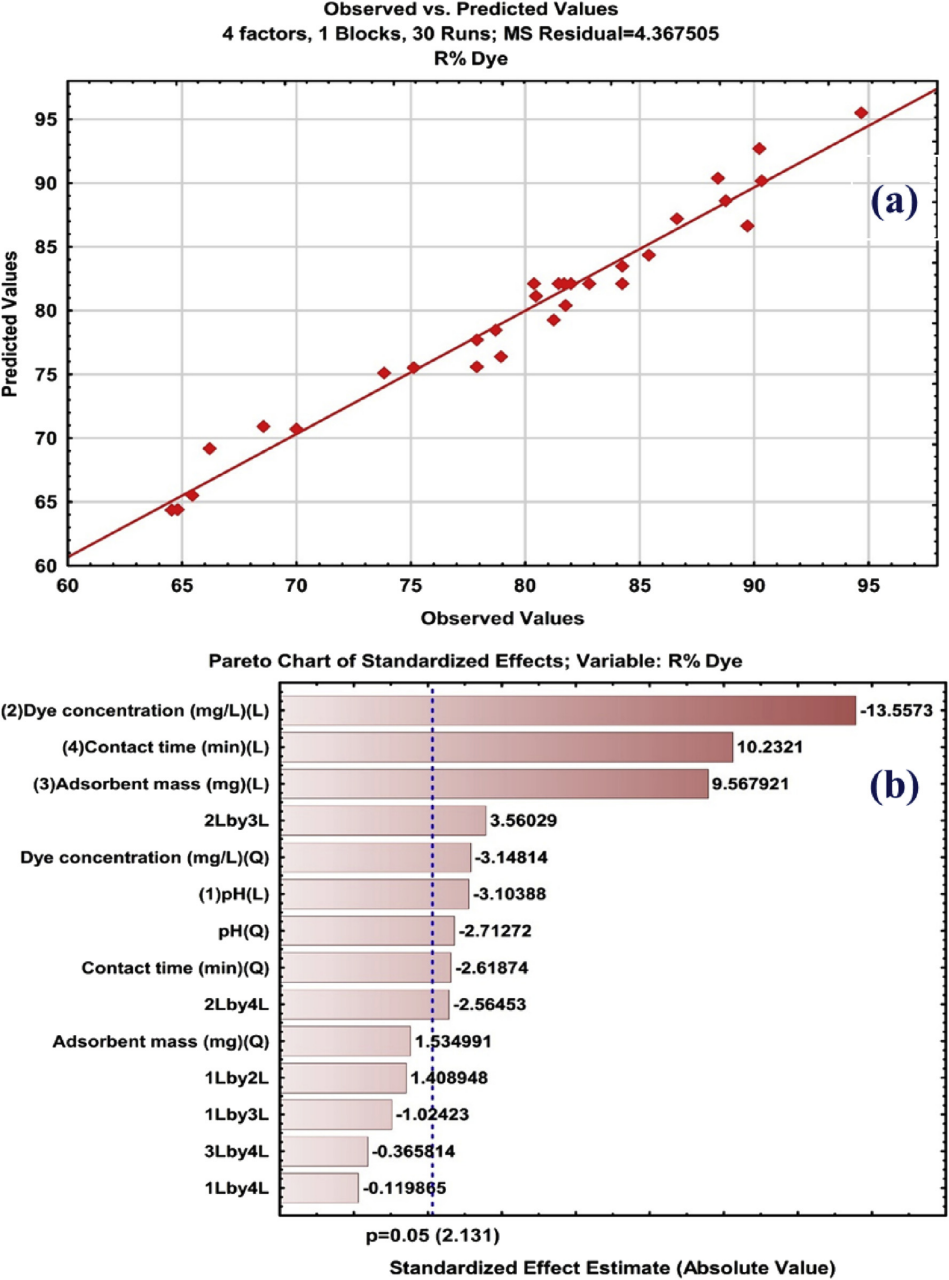
(a) Plot of actual response versus predicted response and (b) Standardized main effect Pareto chart for the removal of CR using CCD.

A linear relationship between the predicted and investigational values of the responses was observed in Fig. 4a with high correlation coefficient that shows the applicability of models. The examination of the outcomes is imagined utilizing consistent Pareto charts (P = 95%) (Fig. 4b). The developed equations model in terms of coded factors is given away in the next equations:

(4) R% Pb@ZnFe_2_O_4_ = 82.09–1.324 X_1_ -5.783 X_2_ +4.082 X_3_ +4.365 X_4_ +1.860 X_2_ X_3_ -1.340 X_2_ X_4_ -1.082 X_12_ -1.256 X_22_ -1.045 X_42_

### 3.4 Response surface plots (RSM)

**Fig. 5:**
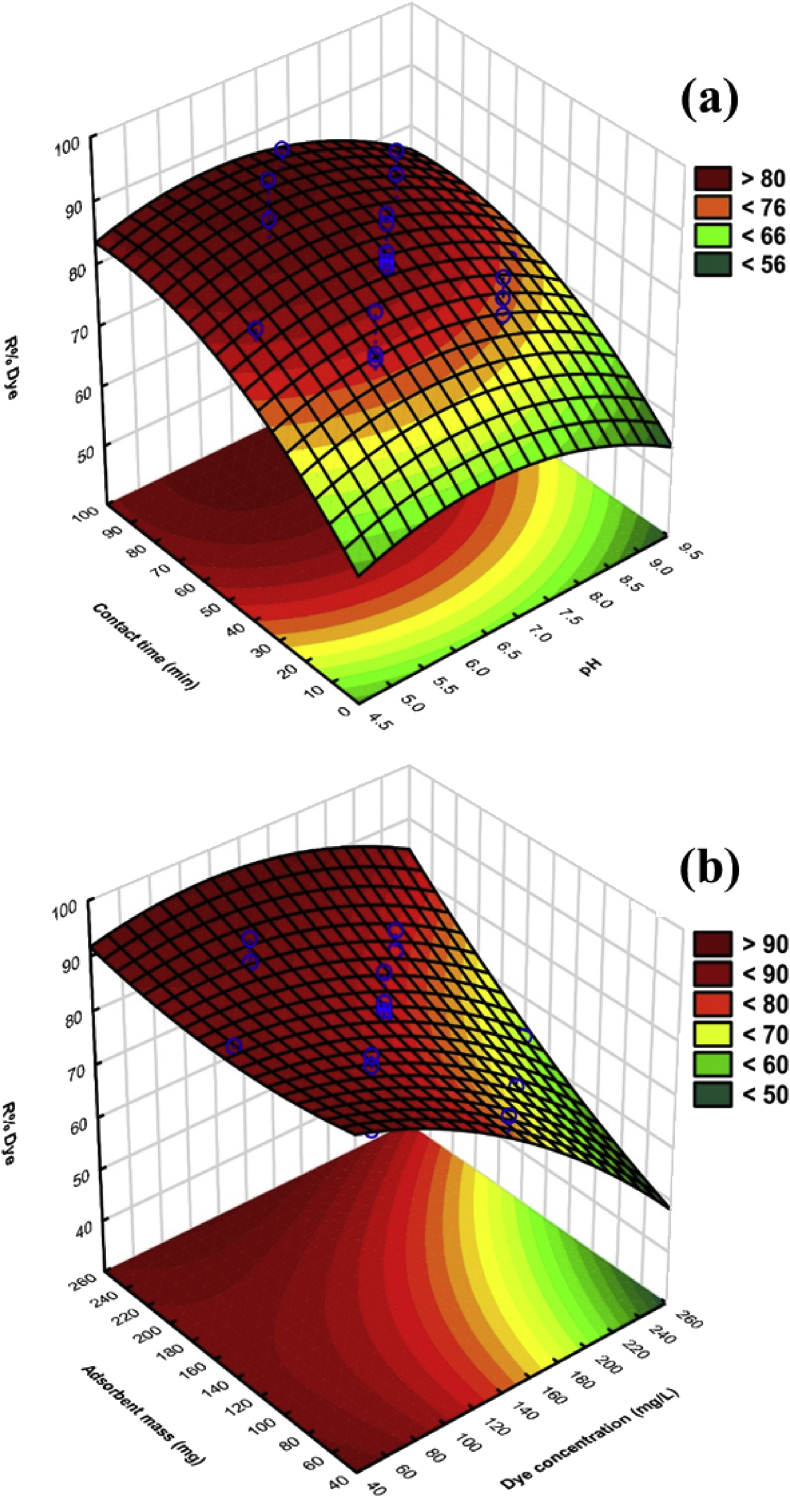
3-D surface plots for interactive effect of (a) pH and contact time, (b) adsorbent mass and initial CR concentration.

3D response surface plots are the most applicable approach applied to indicate the combined effects and identify the main interactions among factors on dye removal [28]. Response surface plots (3D) were applied to demonstrate the collective property and recognize the main interactions among variables on % removal of CR dye by Pb@ZnFe_2_O_4_ adsorbent. In this experiment, typically two 3D RSM plots are revealed in Fig. 5. The 3-D plot (Fig. 5a) illustrates the interaction of the independent variables (contact time and pH) on the response process. According to the 3D plot (Fig. 5a), the adsorption efficiency increased with the increase of contact time under adsorbent process, while a higher pH led to better removal of CR dye. Fig. 5b shows a response surface plot of the removal CR, as dependent on adsorbent mass and the dye concentration. Commonly the adsorbent weight effect on the removal CR illustrate that the increasing in magnitude of adsorbent lead to quick CR adsorption that attributed to greater specific surface area at lower adsorbent mass that inferable from unbalancing the dye atoms toward vacant sites. It is seen that the CR removal decreases by raising the dye concentration, which is owing to the fall in the proportion of available surface area adsorption sites to the CR molecules [27].

### 3.5 Effect of contact time

To optimize the agitation time to accomplish equilibrium, the consequence of agitation time of the adsorption of CR dye was investigated at various contact times (5–120 min) at 30 mg L^−1^ adsorbate solution onto Pb@ZnFe_2_O_4_ at optimum pH. At an optimum pH value, adsorbent added in a 50 ml of 30 mg L^−1^ colored solution. Absorbance of colored solutions at λ_max_ was calculated at different time's intervals. The results are shown in Fig. 5a. One can distinguish that it requires short equilibrium time and therefore it indicates high tendency of CR for rapid migration and diffusion to the Pb@ZnFe_2_O_4_ surface. Obtained result shows that adsorbed CR increased as the time increased and reached the diffusion level after 60 min. However, further increasing the time there was no noteworthy impact on the adsorption of CR, prolonging that a dynamic equilibrium state was reached. This is due to the fact that initially more accessible active sites for the adsorption process but as soon as the progression started the active sites unavailable because of occupying the adsorbate molecules, for this reason after certain time it becomes saturated. At elevated time, there was non-huge difference in expulsion % because of moderate pore dispersion or immersion of adsorbent [29, 30]. Therefore the 60 min period as the optimum agitation time was chosen for further study. As it is obvious, ≥ 90% of CR adsorption achieved in 60 min and thereafter the adsorption rate was slow so that up to 99% of CR removal occurs at 90 min for 0.2 g (200 mg) of adsorbent at 30 mg L^−1^ CR concentration.

### 3.6 Effect of the amount of adsorbent/adsorbate

The effect of Pb@ZnFe_2_O_4_ dosage was studied at RT and at optimum pH. To explore the influence of adsorbent quantity on the system, experiments were conducted varying the adsorbent dosage from 0.05 g/L to 0.20 g/L, keeping the initial CR concentration fixed at 200 mg L^−1^ and the results have been depicted in Fig. 5b. It seems that, the percentage adsorption of dye increased by raising the amount of Pb@ZnFe_2_O_4_ as a result of the increase in surface area as well as binding centers. Extent of adsorption is slowed down after 150 mg L^−1^dosage because of the fact that although there is abundance of active sites but there is lack of dye in the solution. High adsorbent dosage increases the CR removal percentage because of very fast adsorption of dye onto the adsorbent surface that turn out a lesser solute concentration in the solution than when adsorbent dosage is lower [29, 31, 32]. The adsorption reached a maximum with 0.20 g/L of adsorbent for CR dye, with highest proportion removal of about 99%.

### 3.7 Optimization condition study

**Fig. 6:**
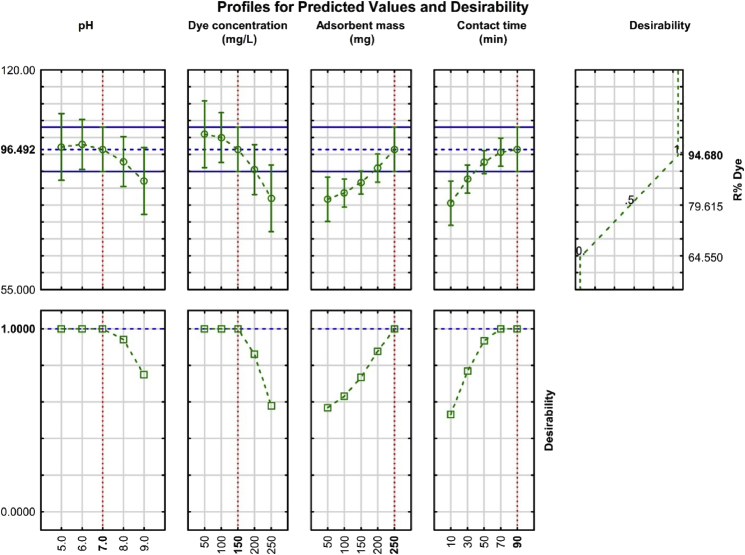
Profiles of predicted values and desirability function for CR dye (Dotted lines indicate optimization values).

The desirable profile responses for removal % of CR dye with Pb@ZnFe_2_O_4_ adsorbent by conveying predicted value shows in Fig. 6. The variable (X_1_-X_4_) in the model was illustrated at the bottom of Fig. 6 (Table T3 in supplement), while their removal percentage is shown in left hand and the desirability close to 1 indicates the most desired conditions. The RSM experiments performed and the 94.8 % of maximum R% of CR was found, while the minimum of 64.55 % were observed for CR. In accordance with this profile, desirability score of 1 (optimum conditions) achieved at following situation: 7 as pH, 150 mg.L^−1^ CR dye concentration and 250 mg as adsorbent mass (Pb@ZnFe_2_O_4_) and 90 min as contact time permit obtaining, 96.49% removal percentage.

In order to achieve appropriate mechanism of adsorption, investigation of kinetic models is required [33]. The kinetic parameters results (Table 1) through taking into consideration correlation coefficients (R^2^ = 0.999) show that the kinetic pseudo-second-order equation be able to predict experimental data. The results confirmed that the experimental and predicted q_e_ was very reasonable closeness. The kinetic data were fitted with pseudo-second-order kinetic model. The intraparticle diffusion plot shows that there are 2 or more variables controlling the rate of the adsorption. The ability of the Pb@ZnFe_2_O_4_ to remove dye from the water is very fast showing considerable potential for real applications. For Investigation of the dispersion mechanism between CR molecule and Pb@ZnFe_2_O_4_, intra-particle diffusion model has been applied and the initial rate of intraparticle diffusion is prearranged by the Supplementary Eq. (7). Some of the authors reported that the only intraparticle diffusion step is rate preventive step, when the plot goes through the origin [34]. In obtained result, the plot does not go through the origin instead; qt against t^1/2^ plot tends to introduce a multi-straight line that demonstrates commitment of at least two stages in the adsorption. Outside surface adsorption is quick, while intraparticle diffusion and equilibrium thought to be rate limitting step. Bigger estimation of the intercept (reflects the boundry layer impact) affirms more prominent contribution of the surface adsorption.

**Table 1:** Kinetic parameters of adsorption of CR onto Pb@ZnFe_2_O_4,_ Conditions: 0.20 g/L adsorbent over 10–200 mg L^−1^of CR dye concentration at optimum conditions of other variables.

Models	Parameters	Parameter values: concentration of dye (mg L-1)					
		10	20	50	100	150	200
First order Log (qe-qt) = log(qe)-(K_1_/2.303)t	K_1_ × 10^2^	4.14	4.14	2.99	3.68	5.76	7.14
	q_e_	0.843	1.149	1.459	1.697	1.787	1.9
	R^2^	0.796	0.929	0.961	0.953	0.869	0.982
Second order t/qt = 1/K_2_qe^2^+ (1/qe)t	K_2_ × 10^2^	1.28	0.65	0.26	0.18	0.16	0.13
	q_e_	10.41	20.833	52.63	100	_1042_.85	200
	R^2^	0.987	0.989	0.998	0.998	0.999	0.999
Intraparticle diffusion: qt = K_diff_ t^1/2^ + C	K_diff_	0.698	1.398	4.015	7.412	10.08	11.27
	C	3.569	7.079	14.73	34.92	58.24	78.57
	R^2^	0.993	0.99	0.891	0.83	0.784	0.72

### 3.8 Adsorption mechanism

**Fig. 7:**
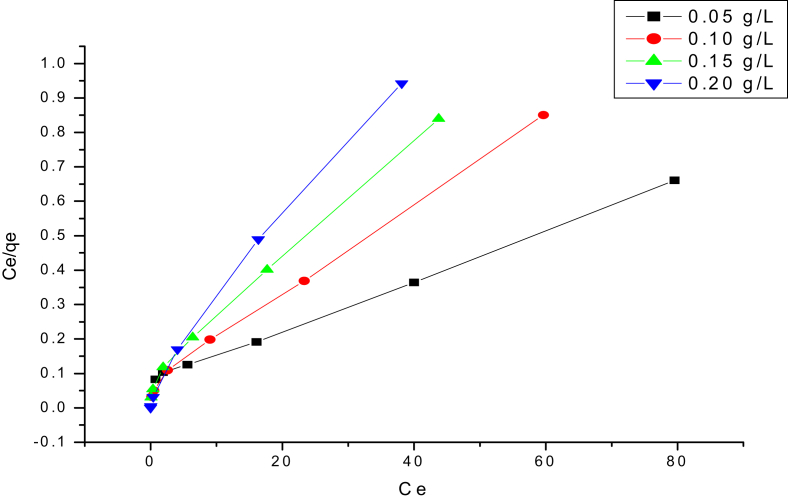
Linear fitting of Langmuir isotherm for four different adsorbent dosages (0.05–0.2 gL^-1^).

The adsorption isotherms provide considerable information for specifying the adsorbent–adsorbate interaction mechanism. The adsorption isotherms were studied by Langmuir, Freundlich, Temkin, Dubinin–Radushkevich and Hurkins-Jura models. These common isotherms used to determine adsorption isotherm parameters to apply linear regression with transformed variables. The equilibrium isotherm for the adsorption including Q_m_, K_a_, K_f_, n, B_1_, K_T_, Q_S_, K, E, A, B and R^2^ are given in Table 2. Among various adsorption isotherm models, the data of CR was better fitted with Langmuir model (Fig. 7) with R^2^ of 0.999, 0.996, 0.993 and 0.999 for 50, 100, 150 and 200 mg/L adsorbent concentration, respectively. The maximum adsorption capacities based on Langmuir model for CR onto Pb@ZnFe_2_O_4_ NCs were found to be 1042.86 mg/g.

(5) Langmuir model = Ce/qe = CeαL/K_L_ + 1/K_L_

**Table 2:** Isotherm constant parameters and correlation coefficients calculated for the adsorption at various concentration of CR (10–200 mg/L), pH = 6 and 120 min contact time onto Pb@ZnFe_2_O_4_.

Isotherm	Equation	Parameter values: Adsorbent conc. (mg/L)				
		Parameters	50	100	150	200
Langmuir	Ce/qe = CeαL/K_L_ + 1/K_L_	q_max_	1042.86	976.92	755.55	641.66
		K_a_	29.41	1.8181	15.625	6.17
		R^2^	0.999	0.996	0.993	0.999
Freundlich	Ln qe = ln K_F_ + (1/n)ln Ce	1/n	0.804	0.752	0.767	0.726
		K_f_	12.41	14.36	15.74	6.073
		R^2^	0.993	0.987	0.985	0.978
Tempkin	qe = B_l_ ln KT + Bl ln Ce	Β_1_	26.11	0.081	0.113	0.239
		K_T_	1.36	64 × 10^7^	24 × 10^6^	29 × 10^8^
		R^2^	0.979	0.962	0.952	0.922
Dubinin and Radushkevich	Ln qe = ln Qs – Kε^2^	Qs	74.36	42.09	29.57	23.46
		K × 10^7^	5	10	70	100
		E	1000	2236	2672	7071
		R^2^	0.765	0.779	0.772	0.809
Hurkins-Jura	1/Qe^2^ = [B/A]-[1/A] logCe	A	250	76.92	34.48	32.25
		B	1.75	1.23	1.103	0.903
		R^2^	0.631	0.657	0.671	0.72

The calculated ΔG° values of this adsorption system are listed (Table 3). The negative ΔG value in the considered temperature range confirms the practicability and spontaneity of the adsorption and the adsorption is more favorable at lower temperature [35]. The negative values of ΔH° for the considered range of temperature support the exothermic nature of the process. The ΔS° corresponds to a reduced in adsorbed species degree of freedom at the solid-solution boundary of the entire adsorption course. The values achieved from Eqs. (4) and (5) are listed (Table 3).

**Table 3:** Thermodynamic parameters for CR adsorption on Pb@ZnFe_2_O_4_

NPs	Temp. (K)	ΔG° (kJ mol^−1^)	ΔH° (kJ mol^−1^)	ΔS° (J mol-1 K^−1^)
50 mg	298	-8.377	-0.033	-0.249
	308	-8.71		
	318	-9.06		
100 mg	298	-1.481	-0.026	-0.149
	308	-2.364		
	318	-3.090		
150 mg	298	-6.810	-0.0166	-0.0831
	308	-7.151		
	318	-7.49		
200 mg	298	-4.509	-0.0083	-0.0415
	308	-4.935		
	318	-5.352		

### 3.9 Reusability of Pb@ZnFe_2_O_4_

The technology or material used for waste treatment should be economical. The reuse study has been done to prove that the current adsorbent has good adsorption ability as well as can be recycled very easily and again gets ready for use. Desorption progression was carried out on nanoparticles by assimilation of dye loaded Pb@ZnFe_2_O_4_ with 2 mL of eluents of 1:1 combination of 0.1 molL^-1^ NaOH and methanol and more than 94% desorption efficiency for them was calculated up to 3^rd^ adsorption–desorption cycle (Supplementary Fig. S7). Therefore, the dye might be desorbed from the encumbered nanoparticles and 1:1 mixture of 0.1 molL^-1^ NaOH and methanol might be used as an operational solution for desorption and reusability studies of Pb@ZnFe_2_O_4_ [36, 37, 38]. So, it affirmed that Pb@ZnFe_2_O_4_ can be renewed easily with 1:1 combination of 0.1 molL^−1^NaOH and methanol solution treatment, and may be used frequently as a competent adsorbent for realistic wastewater treatment.

### 3.10 Real wastewater decontamination study

**Fig. 8:**
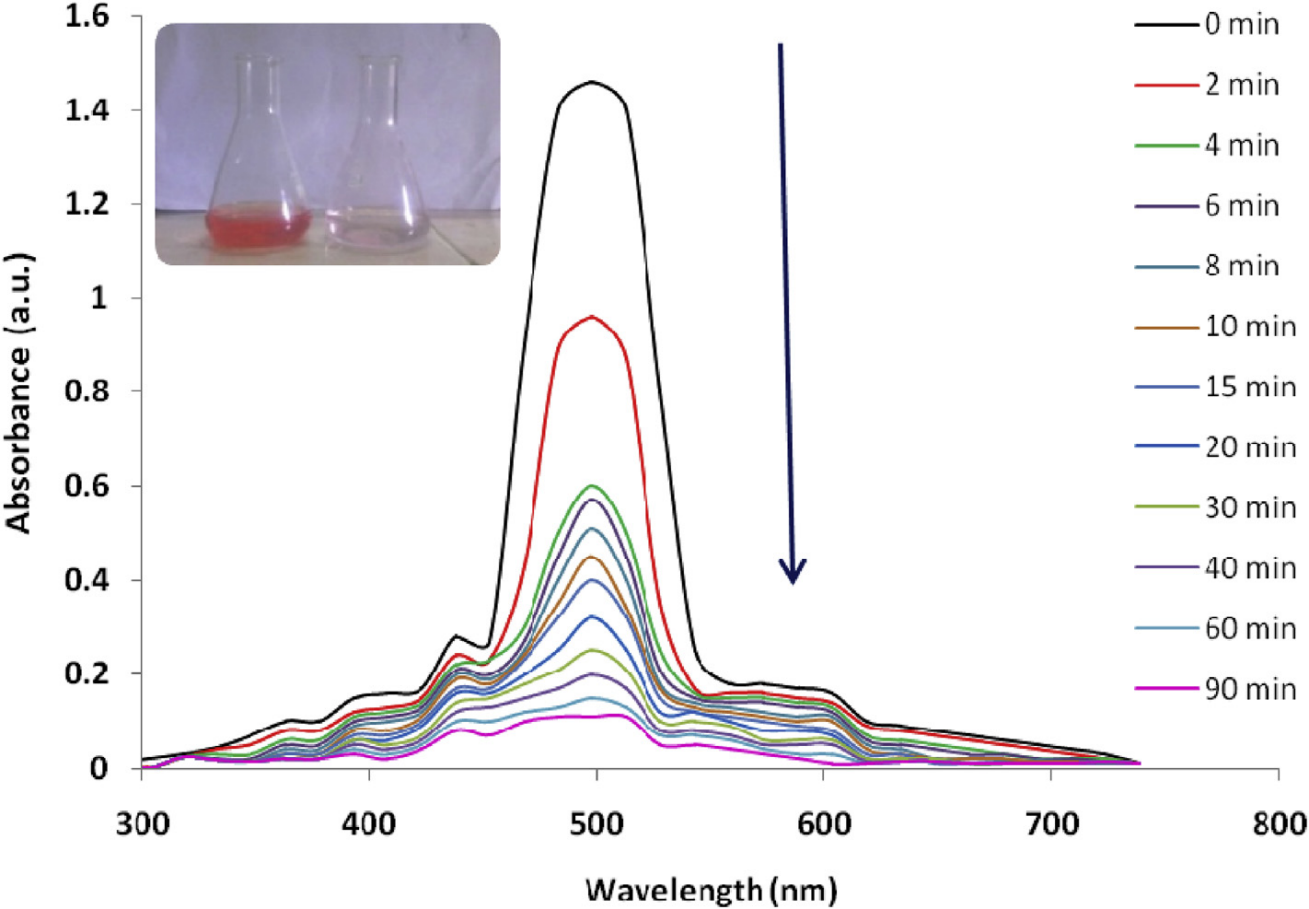
Real Sample analysis.

In conformity with the previously adopted optimal parameters and conditions for the decontamination of CR dye, the ability to treat a real wastewater system containing this dye, has been further investigated. Real waste water sample has been collected from local industrial area and diluted. The industrial effluent treated at obtained optimum conditions. The mixture has been analyzed spectrophotometrically. The outcome exposed that the real sample has been decolorized up to 89% after 90 min. Peak observed at 498 nm (*λ* max for CR) at opening time (0 min) shrink without any shift in λ_max_ up to entire decolourization of the mixture (Fig. 8).

### 3.11 Comparison of Pb@ZnFe_2_O_4_ with other adsorbents

A comparison of performance of CR adsorption capability of formerly reported adsorbents is presented in Table 4. The results indicate that adsorption capacity for Pb@ZnFe_2_O_4_ is preferable and superior and shows reasonable removal performance for CR. The adsorption of Pb@ZnFe_2_O_4_ are extremely dependent on the structure of the sorbate as a probe and the adsorptive include networks, defects, and functional groups.

**Table 4:** Comparison of the adsorption capacity of some adsorbents towards CR dye.

Adsorbent	Q_max_ (mg/g)	References
NiO Nanoparticle	39.7	[39]
Ni_0.6_Fe_2.4_O_4_	72.73	[21]
Cashew nut shell	5.18	[40]
Kaolin	5.44	[41]
Na Bentonite	35.84	[41]
Acid-treated pine cone	40.19	[42]
Raw pine cone	19.18	[42]
Tamarind fruit shell	10.48	[43]
Magnetically modified fodder yeast cell	49.7	[44]
Chitosan/montmorillonite nanocomposite	54.52	[45]
Nickel(II) oxide	534.8	[46]
Nickel(II) hydroxide	384.6	[46]
Ni/Mg/Al layered double hydroxides	1250	[47]
hierarchical porous zinc oxide	334	[48]
Pb@ZnFe_2_O_4_	1042.86	Present Study

## 4 Conclusion

In the present work, Pb@ZnFe_2_O_4_ with rough surfaces was synthesized by simple co-precipitation method. The varieties of the size, microstructure and morphology in as-combined sample are affirmed by XRD, SEM and EDX techniques. The adsorbent can be used for expulsion of CR from waste water. The CCD model was effectively connected to examine the interactive impacts of adsorption factors and enhance the adsorption. The greatest adsorption effectiveness for evacuation with Pb@ZnFe_2_O_4_ at pH: 7.0, adsorbent mass: 250 mg, starting fixation CR dye: 150 mg L^−1^ at 90 min evacuation is around 96.49 %. The adsorption conduct of CR onto the prepared Pb@ZnFe_2_O_4_ was methodically researched, which was observed to be spontaneous, exothermic and obey pseudo-second order rate equation. Furthermore, the Langmuir isotherm is better than the Freundlich isotherm to fit the exploratory information. The monolayer adsorption limit of Pb@ZnFe_2_O_4_ for CR is 1042 mg g^−1^. Together with the thermodynamic parameters, our outcomes demonstrate that chemisorption assume the overwhelming job in the adsorption procedure. The blended Pb@ZnFe_2_O_4_ can be utilized as a productive and recyclable adsorbent for the expulsion of CR from aqueous media.

## Declarations

### Author contribution statement

Sanjay Attarde: Conceived and designed the experiments; Analyzed and interpreted the data.

Ganesh Jethave: Performed the experiments; Wrote the paper.

Umesh Fegade: Conceived and designed the experiments; Wrote the paper.

Sopan Ingle: Contributed reagents, materials, analysis tools or data. Mehrorang Ghaedi, Mohammad Mehdi Sabzehmeidani: Analyzed and interpreted the data.

### Funding statement

This work was supported by the 10.13039/501100001412Council of Scientific & Industrial Research (CSIR), India, for financial support under CSIR-SRF (Direct) Scheme fellowship awarded to first author Mr. Ganesh Jethave (Ack. No.122066/2K17/1).

### Competing interest statement

The authors declare no conflict of interest.

### Additional information

No additional information is available for this paper.

## Appendix A Supplementary data

Suppliment_V2 Suppliment_V2
