# Corrigendum to “Exploration of the adsorption capability by doping Pb@ZnFe_2_O_4_ nanocomposites (NCs) for decontamination of dye from textile wastewater” [Heliyon Volume 5, Issue 9, September 2019, e02412]

**DOI:** 10.1016/j.heliyon.2020.e03367

**Published:** 2020-02-19

**Authors:** Ganesh Jethave, Umesh Fegade, Sanjay Attarde, Sopan Ingle, Mehrorang Ghaedi, Mohammad Mehdi Sabzehmeidani

**Affiliations:** a School of Environmental and Earth Sciences, KBC North Maharashtra University, Jalgaon, MS, India; b Bhusawal Arts, Science and P. O. Nahata Commerce College, Bhusawal, MS, India; c Chemistry Department, Yasouj University, Yasouj, Iran; d Chemical Engineering Department, Yasouj University, Yasouj, Iran

In the original published version of this article, an incorrect version of figure 2a was displayed. This has now been corrected as shown below. The author apologizes for this mistake. Both the HTML and PDF versions of the article have been updated to correct the error.

**Fig. 2:**
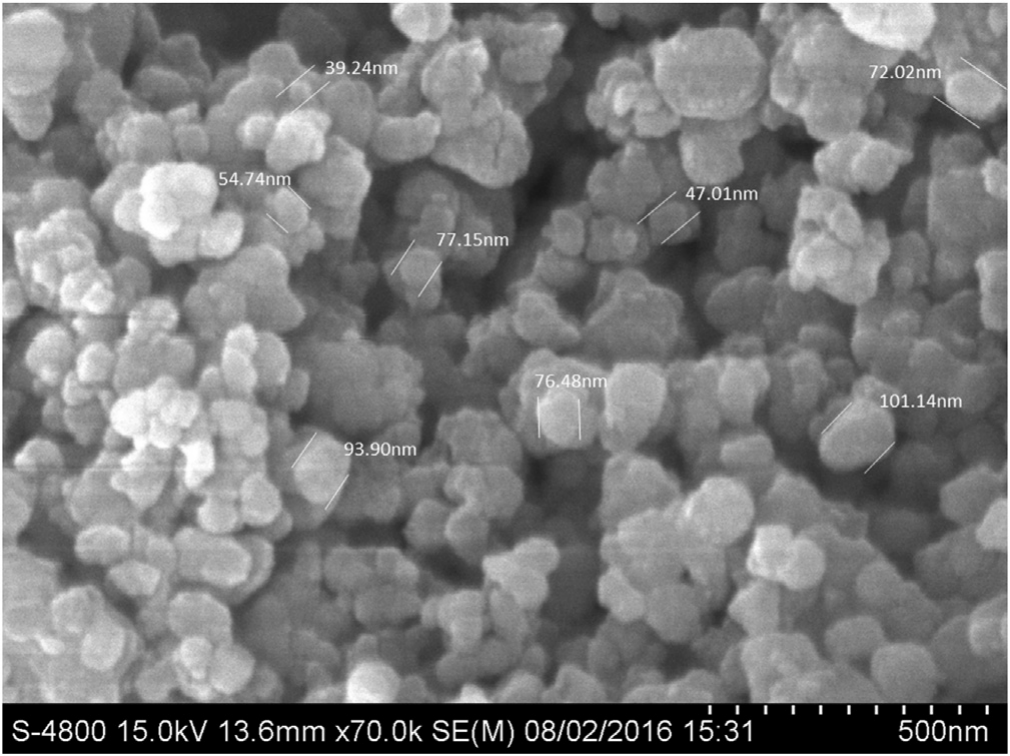
(a) SEM Images of Pb@ZnFe^2^O^4^ average particle size 70.21 nm at low magnification.

