# Supplementary material for: Exploration of the adsorption capability by doping Pb@ZnFe2O4 nanocomposites (NCs) for decontamination of dye from textile wastewater
Source: Heliyon. 2019 Sep 19;5(9):e02412. doi: 10.1016/j.heliyon.2019.e02412 (PMC6819840; doi:10.1016/j.heliyon.2019.e02412)
Supplement: Suppliment_V2 [file mmc1.doc]

**Exploration of the adsorption capability by doping Pb@ZnFe_2_O_4_ Nanocomposites (NCs) for decontamination of textile dye from wastewater**

Ganesh Jethave^a^, UmeshFegade^b^, Sanjay Attarde^a*^, Mehrorang Ghaedi^c^, Mohammad Mehdi Sabzehmeidani^d^

^a^School of Environmental and Earth Sciences, North Maharashtra University, Jalgaon, MS, India.

^b^Bhusawal Arts, Science and P.O.Nahata Commerce College, Bhusawal, MS, India.

^c^Chemistry Department, Yasouj University, Yasouj 75918-74831, Iran.

^d^Chemical Engineering Department, Yasouj University, Yasouj, Iran.

Corresponding author *Email ID: [sb.attarde@yahoo.co.in](mailto:sb.attarde@yahoo.co.in)

* E-Mail: [m_ghaedi@yu.ac.ir](mailto:m_ghaedi@yu.ac.ir) , Tel & fax: +98-74-33223048

Supplement

**2.4 Response surface design**

The response surface methodology (RSM) is useful to evaluate and recognize the significant variables and interactions effects of them by an appropriate model to predict the relationship among controllable input parameters [1-4].In the present study, four factor (pH, dye concentration (mg L^-1^), adsorbent dose (mg) and contact time (min)) was used to investigate the effects of parameters into five levels CCD (low, central and high).The model to study the dependency of removal efficiency to variables and predict their real behavior (Table T1). A 30 experiments design matrix corresponds the responses of dye are shown in Table T1 that it gives beneficial information about applicability of the proposed model. The mathematical relationship between independent variables can be approximated by the second order polynomial model.

**Table T-1:** Matrix for the central composite design (CCD)

| Levels | | | | | Factors |
| --- | --- | --- | --- | --- | --- |
| +α | -α | High(+1) | Central(0) | Low (-1) |
| 9.0 | 5.0 | 8.0 | 7.0 | 6.0 | X_1_:pH |
| 250 | 50 | 200 | 150 | 100 | X_2_: Dye concentration (mg L^-1^) |
| 250 | 50 | 200 | 150 | 100 | X_3_: Adsorbent dose (mg) |
| 90 | 10 | 70 | 50 | 30 | X_4_: Contact time (min) |

**Fig. S1:** Molecular Structure of CR Dye


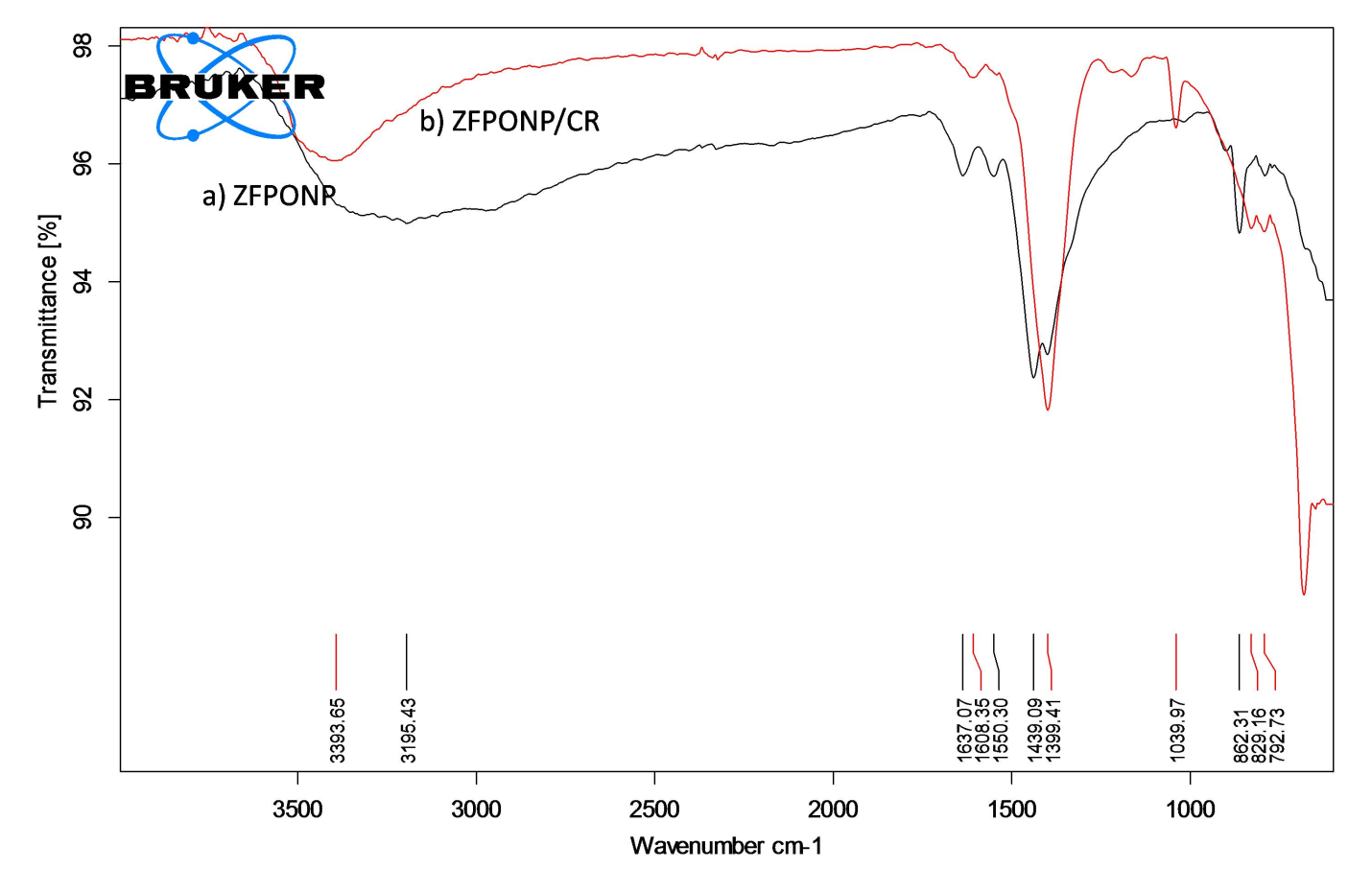


**Fig. S2:** FT-IR spectra of Pb@ZnFe_2_O_4_ (a), CR coated- Pb@ZnFe_2_O_4_ (b).

**THEORY**

**Adsorption isotherm:** Many adsorption isotherm models are used to assess the applicability of the proposed nanoparticles i.e. Pb@ZnFe_2_O_4_ for adsorption of dye. The isotherm parameters and the underlying thermodynamic assumptions of the equilibrium often give some information regarding the adsorption mechanism, surface properties, maximum adsorption capacity and affinity of the adsorbent. Among all of the models, to suggest the adsorption isotherm, five commonly used isotherm models, such as the Langmuir, Freundlich, Temkin, Dubinin-Radushkevich and Hurkins-Jura applied to predict the equilibrium parameters and molecular adsorption at interfaces and also to elucidate CR dye- Pb@ZnFe_2_O_4_ interaction [1-8].

**Langmuir Isotherm:** Langmuir isotherm assumes monolayer coverage of adsorption of each molecule onto homogeneous active sites on adsorbents without any lateral interaction between adsorbed dye molecules on neighboring sites. According to this model, there are finite numbers of active centers with the equivalent adsorption energy on the adsorbent surface [5]. This model has created good agreement with experimental data and can be expressed as in supplementary equation 1.

$\frac{C_{e}}{q_{e}}=C_{e}\left(\frac{\alpha_{L}}{K_{L}}\right)+\left(\frac{1}{K_{L}}\right)$ (1)

Where,

qe = Adsorption capacity (mg/g),

Ce = CR concentration (mg/L) at equilibrium;

1/K_L_ and (α_L_/K_L_) q_max_ = Langmuir constants determined by the intercept and slope of the linear plot of Ce/q versus Ce, respectively.

**Freundlich isotherm:** To describe solid-liquid adsorption, the Freundlich isotherm model (supplementary Eq. 2) is widely applied. Unlike the Langmuir isotherm model, this model recognizes a multilayer adsorption onto heterogeneous adsorption surfaces with non-equivalent energy binding sites and interactions between adsorbed molecules [6].

$\ln q_{e}=\ln K_{f}+\frac{1}{n}(\ln C_{e})$ (2)

Where

K_f_ = Freundlich adsorption constant (l/mg) and

1/n = Adsorption intensity. These parameters can be calculated from the intercept and slop of the plot of log qe versus log Ce, respectively.

**Adsorption kinetics:** The kinetics for CR dye adsorption on Pb@ZnFe_2_O_4_ was evaluated by fitting the experimental data with the pseudo-first-order and pseudo-second-order kinetic models. To investigate and establish the mechanism of adsorption, the rate determining steps particularly potential rate-controlling step, for CR dye adsorption, experimental data were further analyzed by the intra-particle diffusion model.

**Pseudo-first-order and Pseudo-second-order model**

The integral form of the pseudo-first-order model generally expressed as in supplementary equation 3.

The adsorption data was then analyzed for pseudo-second-order mechanism, described by supplementary equation 5.

$\log\left(q_{e}-q_{t}\right)=\log\left(q_{t}\right)-\left(\frac{K_{1}}{2.303}\right)t$ (3)

Where

qe and qt = Amount of dye adsorbed (mg/g) at equilibrium time and at any time t respectively, K_1_ = Adsorption first-order rate constant (min^-1^) and

t = Contact time (min).

The adsorption rate constantK_1_ was calculated from the plot of log (qe- qt) vs. t.

**Pseudo-second-order model**

$\frac{dq_{t}}{q_{t}}=K_{2}\left(q_{e}-q_{t}\right)^{2}$ (4)

Where,

K_2_ = Pseudo-second-order rate constant (g/mg min).

Integrating and applying boundary conditions t =0 to t= t and q= 0 to q = qt gives

$\frac{t}{q_{t}}=\left(\frac{1}{K_{2}q_{e}^{2}}\right)+\left(\frac{1}{q_{e}}\right)t$ (5)

A plot between t/qt versus t gives the value of the constants K_2_ (g/mg min) and also qe (mg/g) can be calculated. The Constant K_2_ is used to calculate the initial adsorption rate h, at t→0, as follows:

$h=K_{2}q_{e}^{2}$ (6)

Thus the rate constant K_2_, initial adsorption rate h and predicted qe can be calculated from the plot of t/qt versus t using Eq. (5).

**Adsorption mechanism**

According to this model (supplementary equation 7) from the shape of qt vs. t^1/2^ plot, the rate-controlling step can be deduced. Rate is controlled only by intra-particle diffusion when a linear plots passing through the origin. On the other hand, when a non-linear or linear plot which does not pass through the origin then we can say that the rate is limited by two or more steps.

$q_{t}=K_{id}t^{\frac{1}{2}}+C$ (7)

Where,

qt = Amount of dye adsorbed at time t,

t^½ =^ Square root of the time and

K_id_ (mg/g min^½^) = Rate constant of intra-particle diffusion.

**Temkin isotherm model [7]**

$q_{e}=B_{1}\ln KT+B_{1}\ln C_{e}$ (8)

Where,

C_e_ = Concentration of dye remain in solution at Equilibrium (mg L^-1^),

q_e_ = Amount of dye adsorbed at equilibrium (mg g^-1^),

B = Temkin constant related to heat of the adsorption (J mol^−1^),

*T* = Absolute temperature (K),

*R* = universal gas constant (8.314 J mol^−1^.K^−1^) and

*K* = Equilibrium binding constant (L mg^−1^).

**D–R model [8]**

$q_{e}=Q_{s}\exp(-B\varepsilon^{2})$ (9)

$\ln q_{e}=\ln q_{s}-B\varepsilon^{2}$ (10)

Where,

B = Constant related to the adsorption energy,

Qs = Theoretical saturation capacity and

ε = Polanyi potential, calculated from Equation (11).

$\varepsilon=RT\ln\left(1+\frac{1}{C_{e}}\right)$ (11)

The values of Qs and B were calculated from the intercept and slope of the plot ln qe versus ε^2^. The mean free energy of adsorption E calculated from B using the following equation 12.

$E=\frac{1}{(2B)^{\frac{1}{2}}}$ (12)

**Fig. S3:** Influence of pH (Adsorbents Pb@ZnFe_2_O_4_ 0.05 g/L; Initial dye conc.: 20 mg/L)


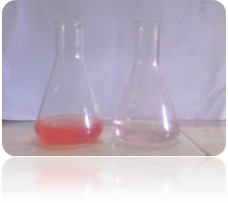


**Fig. S4:** Effect of the amount of adsorbent/adsorbate (Adsorbent Pb@ZnFe_2_O_4_; 0.05 g L^-1^ to 0.20

g L^-1^; Initial dye conc.: 10 - 200 mg L^-1^, pH=6) **Inset Fig**.: The CR dye solution before and after adsorption.

**Fig. S5:** Intra particle diffusion plot on different initial dye concentrations.

**Fig. S6:** Separation factor for the adsorption of Congo red onto Pb@ZnFe_2_O_4_ at 25 °C.


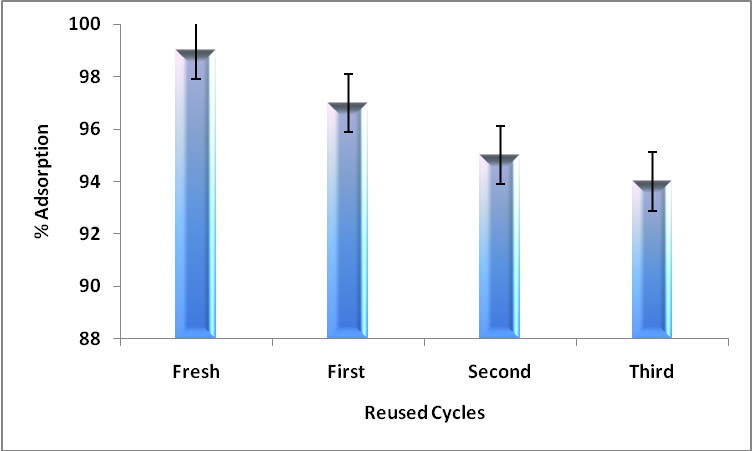


**Fig. S7:** Influence of regeneration of Pb@ZnFe_2_O_4_ by alkali wash on adsorption capacity

**Adsorption Kinetics**

To study the adsorption rate of dye on the prepared Pb@ZnFe_2_O_4_, experimental data were fitted in pseudo first and pseudo second order kinetic models. For this study, CR sample solutions were shaken by means of rotary shaker and carried out for 90 min with the Pb@ZnFe_2_O_4_. In order to know the applicability of these two models, six initial dye concentrations viz. 10, 20, 50, 100, 150 and 200 mg L^-1^ were used. The adsorption capacity of Pb@ZnFe_2_O_4_ was enhanced rapidly in the initial time, and then slowed down, which finally level out. In this case of Pb@ZnFe_2_O_4_, 90 min was enough to attain adsorption equilibrium; the rapid adsorption of CR by the Pb@ZnFe_2_O_4_ suggests that the adsorptive sites for CR exist on the entire structure of the nanoparticles, which are easily accessible to the anionic species in CR [1-3].

The kinetics of CR dye adsorption onto Pb@ZnFe_2_O_4_ was explored using pseudo-second order, pseudo-first order kinetic and intra-particle diffusion model. The various parameters were calculated from the plots of the kinetic model equations and summarized in Table 1. It may be seen that the pseudo-second order kinetic model (Fig. S8) describes the best adsorption kinetics. The linear fits of the kinetics results reflect that pseudo second order model exhibits higher R^2^ (0.99) value, compared to pseudo-first order model. Another alternative method for kinetic evaluation of an adsorption process is intra-particle diffusion model. According to conditions and equation presented in Table 1, intraparticle diffusion said to be rate controlling step when respective plot (plot of qt versus t^1/2^) should give only one line and the rate constant K_diff_ was evaluated from the slope of the line supplementary Fig. S5. Since the line did not pass through the origin, it indicates that the intra-particle diffusion was not the rate-controlling step [1-4]. The two kinetic models equations are given in supplementary eq. 3 and 5.

**Fig. S8:** Pseudo-second order kinetics of CR dye adsorption. Conditions: mass of adsorbent 200 mg, pH, 6.0; initial CR concentration 10, 20, 50, 100,150 and 200 mg L^-1^; temperature, 298 K

**Equilibrium studies**

The equilibrium isotherm of a specific adsorbent represents its adsorptive capacity as well as characteristics and is very important to design adsorption method. The adsorption isotherm explains the distribution of adsorbate molecules between the liquid phase and solid phase when the adsorption attained equilibrium. The Langmuir model assumes that a monolayer adsorption depends on the active centers on the adsorbent surface and there is no interaction between adsorbed species. And, all number of adsorption sites are identical and equivalent energetically. While the Freundlich model assumes that the adsorption occurs on a heterogeneous adsorbent surface[9, 10]. Fig.7 in the manuscript showing that, the experimental data fit the Langmuir adsorption isotherm well, having correlation coefficients R^2^ = 0.999. The parameters of the Langmuir model are listed in Table 2. Pb@ZnFe_2_O_4_ showing the monolayer saturated adsorption capacity (maximum adsorption capacity) i.e. q_max_=1042 mg g^-1^. Under similar experimental conditions Table 2 summarizes the adsorption capacity of different amounts of adsorbents for Congo red. It shows Pb@ZnFe_2_O_4_ have high adsorption capacity. The q_max_ could be calculated from the linear form of Langmuir isotherm as shown in the equation 1.

The Freundlich isotherm model (nonlinear model) assisted in explanation of multilayer adsorption with interaction between adsorbed molecules. The Freundlich isotherm model is represented by of exponential adsorption of targeted species on heterogeneous surfaces [6]. The Freundlich isotherm can be simplified to the equation13.

$q_{e}=K_{f}C_{e}^{\frac{1}{n}}$ (13)

The linear form of the Freundlich adsorption isotherm is equation 4. Respective parameters and constants values for this model at various amount of adsorbent were shown in Table 5. As compared to the Langmuir model the correlation coefficients (R^2^ = 0.993) of this model indicate that the Freundlich model has lower efficiency. Therefore, it can be concluded that the CR dye adsorption on the Pb@ZnFe_2_O_4_ is possibly a chemical adsorption (chemisorption) process. The heat of the adsorption and the adsorbent–adsorbate interaction was explained and evaluated by using Temkin isotherm model (supplementary Eq. 8) [34].

A Temkin constants (Table 2) was lower but comparable with Langmuir, Freundlich value and the correlation coefficients R^2^ obtained from Temkin model were also comparable to that obtained from Langmuir and Freundlich equations, which elucidate the applicability of Temkin model to the adsorption of CR onto Pb@ZnFe_2_O_4_ [7].

The Dubinin and Radushkevich (D–R) model was applied to estimate the porosity, free energy and the characteristics of adsorbents[8-10]. It is also useful to find out the nature of the adsorption processes as either physical or chemical. The D–R isotherm dose not assumes a homogeneous or heterogeneous surface or constant adsorption potential. The D–R model has shown in the supplementary Eq. (9) and its linear form can be shown in supplementary Eq. (11).

The positive correlation between E and mass of adsorbent shows higher dye adsorption tendency onto adsorbent surface. The constants of D–R model calculated values (Table 2) shows that the saturation adsorption capacity was in the range of 74-23 (mg g^−1^) and the values of E were between 1000 and 7071 Jmol^-1^ for Pb@ZnFe_2_O_4_ but as R^2^ value is low as compared to Langmuir isotherm the D-R model is not best fitted. The Hurkins-Jura (H-J) adsorption isotherm can be expressed as [1-8].

$\frac{1}{q_{e}^{2}}=\left[\frac{B}{A}\right]-\left[\frac{1}{A}\right]\log C_{e}$ (14)

Where,

Ce = Equilibrium concentration (mg L^-1^),

qe = Amount of dye adsorbed onto the adsorbent (mg g^-1^),

A and B are the isotherm constants.

The constants A and B can be calculated from the plot of 1/q_e_^2^ versus log Ce and the values are tabulated in Table 2. Table 2 listed the parameters of the Langmuir, Freundlich, D-R, Temkin and H-J models along with the regression coefficients (R^2^). The R^2^ values in Table 2 reveal that the Langmuir isotherm fits the experimental results better than remaining four models, involving that the adsorption of CR onto Pb@ZnFe_2_O_4_ follows the mechanism of monolayer adsorption (chemisorption) on a homogenous surface. Similarly shows that the maximum adsorption capacity of Pb@ZnFe_2_O_4_ for CR is 1042 mg g^-1^.

**Separation Factor**

The adsorption efficiency of the process in terms of the dimensionless quantity (R_L_) was also calculated using following equation:

$R_{L}=\frac{1}{(1+\alpha_{L}C_{0})}$ (15)

Where,

α_L_ = Langmuir isotherm constant and

Co = Initial concentration of CR.

R_L_ values within the range 0 <R_L_< 1 indicate favorable adsorption [1-5]. In this study, R_L_ value of Pb@ZnFe_2_O_4_ for the initial CR concentration of 30 mg L^-1^, obtained as 0.83, indicate favorable adsorption of CR onto NPs (Supplementary Fig. S6).

**Adsorption Thermodynamic parameters**

The investigation of effect of reaction temperature on this adsorption process has done in the temperature range of 298 to 318 K. In this experiment, initial CR concentration, adsorbent dose and contact time were varied from 10-200 mg L^-1^, 0.05-0.2 g L^-1^ and 5-60 min respectively, keeping the solution pH constant. The important thermodynamic variables, such as change in Gibb's free energy (ΔG°), change in entropy (ΔS°) and change in enthalpy (ΔH°),were computed by the following expressions and presented in Table 3. The values of ΔH° and ΔS° can be calculated from the slope and intercept of the graph of ln Kc Vs 1/T by using the following equation [1-5].

$\ln K_{c}=\Delta S^{\circ}/R-\Delta H^{\circ}/RT$ (15)

Where,

K_c_ = Equilibrium constant,

R =Ideal gas constant (8.314 Jmol^-1^K^-1^) and

T = Temperature in Kelvin.

The value of ΔG° for specific adsorption was calculated from the basically thermodynamic equation:

$\Delta G^{\circ}=RT\ln K_{c}$ (16)

$K_{c}=\frac{q_{e}}{C_{e}}$ (17)

**Analysis of central composite design (CCD)**

Central composite design was used for optimization of variables including the removal percentage of the CR by Pb@ZnFe_2_O_4_ and respective results were shown in Table T2 and the statistical significance of quadratic model was predicted by the ANOVA based on CR as response (Table T3). The model allows evaluating the statistical importance of all the terms from ANOVA was tested by the F-value and the P-value. The result revealed that the F-value of model for CR removal with Pb@ZnFe_2_O_4_ is 31.24. The Lack of Fit F-value of the model for removal of CR dye by Pb@ZnFe_2_O_4_ adsorbent is 3.32. It could be seen from Table 3 that the linear coefficients (X1, X2, X3 and X4), interaction term coefficients (X2X3 and X2X4) and the quadratic term coefficients (X12, X22 andX42) and were significant due to the P-values was smaller than 0.05.

The higher value for determination coefficient (R^2^) value 0.9668 is goodness indication that more than 96.6% of the variations and the adjusted determination coefficient (Adj-R^2^) value more than 0.935. The predicted determination coefficient (pred-R^2^) value of more than 0.827 was in agreement with R^2^ and adj-R^2^ that illustraed in Table T3 and was used to predict good response. Adequate precision of the present model for removal of CR by Pb@ZnFe_2_O_4_ is 21.06 that the response is greater than 4 and indicates the adequacy of the signal.

**Table T2: Values of the independent variables in CCD matrix and observed results**

| Run | Factors | | | | R% Dye | | |
| --- | --- | --- | --- | --- | --- | --- | --- |
|  | X1: pH | X2: Dye concentration (mg L^-1^) | X3: adsorbent dose (mg) | X4: Contact time (min) | Observed a | Predicted b | Residual c |
| 1 | 6.0 | 100 | 100 | 30 | 78.700 | 78.450 | 0.249 |
| 2 | 8.0 | 100 | 100 | 30 | 75.122 | 75.530 | -0.404 |
| 3 | 6.0 | 200 | 100 | 30 | 64.550 | 64.370 | 0.179 |
| 4 | 8.0 | 200 | 100 | 30 | 64.800 | 64.390 | 0.409 |
| 5 | 6.0 | 100 | 200 | 30 | 85.390 | 84.350 | 1.044 |
| 6 | 8.0 | 100 | 200 | 30 | 81.250 | 79.280 | 1.969 |
| 7 | 6.0 | 200 | 200 | 30 | 77.870 | 77.710 | 0.163 |
| 8 | 8.0 | 200 | 200 | 30 | 77.870 | 75.590 | 2.284 |
| 9 | 6.0 | 100 | 100 | 70 | 88.420 | 90.370 | -1.948 |
| 10 | 8.0 | 100 | 100 | 70 | 86.620 | 87.190 | -0.572 |
| 11 | 6.0 | 200 | 100 | 70 | 68.550 | 70.930 | -2.379 |
| 12 | 8.0 | 200 | 100 | 70 | 69.990 | 70.700 | -0.708 |
| 13 | 6.0 | 100 | 200 | 70 | 94.680 | 95.500 | -0.819 |
| 14 | 8.0 | 100 | 200 | 70 | 90.340 | 90.180 | 0.157 |
| 15 | 6.0 | 200 | 200 | 70 | 84.240 | 83.500 | 0.740 |
| 16 | 8.0 | 200 | 200 | 70 | 80.470 | 81.130 | -0.659 |
| 17 | 5.0 | 150 | 150 | 50 | 81.760 | 80.410 | 1.348 |
| 18 | 9.0 | 150 | 150 | 50 | 73.840 | 75.120 | -1.275 |
| 19 | 7.0 | 50 | 150 | 50 | 88.760 | 88.640 | 0.125 |
| 20 | 7.0 | 250 | 150 | 50 | 65.450 | 65.500 | -0.052 |
| 21 | 7.0 | 150 | 50 | 50 | 78.930 | 76.380 | 2.550 |
| 22 | 7.0 | 150 | 250 | 50 | 90.230 | 92.710 | -2.477 |
| 23 | 7.0 | 150 | 150 | 10 | 66.200 | 69.180 | -2.984 |
| 24 | 7.0 | 150 | 150 | 90 | 89.700 | 86.640 | 3.057 |
| 25 (C) | 7.0 | 150 | 150 | 50 | 81.700 | 82.090 | -0.393 |
| 26 (C) | 7.0 | 150 | 150 | 50 | 82.000 | 82.090 | -0.093 |
| 27 (C) | 7.0 | 150 | 150 | 50 | 84.230 | 82.090 | 2.137 |
| 28 (C) | 7.0 | 150 | 150 | 50 | 82.800 | 82.090 | 0.707 |
| 29 (C) | 7.0 | 150 | 150 | 50 | 81.450 | 82.090 | -0.643 |
| 30 (C) | 7.0 | 150 | 150 | 50 | 80.380 | 82.090 | -1.713 |

(C): Center point

a Experimental values of response.

b Predicted values of response by RSM proposed model.

c Difference between the actual and predicted values for each point in the design.

**Table T3:** ANOVA evaluation of linear, quadratic, interaction terms and regression of coefficients for response variable

| **Source of**  **variation** | **Sum of**  **square** | **Degree of freedom** | **Mean**  **square** | **F-value** | **P-value** | **Status** |  | **Regression coefficients** | |
| --- | --- | --- | --- | --- | --- | --- | --- | --- | --- |
|  |  |  |  |  |  |  |  | **Factor** | **Coefficient Estimate** |
| Model | 1910.266 | 14 | 136.45 | 31.24 | < 0.0001 | Significant |  | Intercept | 82.09 |
| X1 | 42.077 | 1 | 42.08 | 9.63 | 0.0073 |  |  | X1 | -1.324 |
| X2 | 802.750 | 1 | 802.75 | 183.80 | < 0.0001 |  |  | X2 | -5.783 |
| X3 | 399.824 | 1 | 399.82 | 91.55 | < 0.0001 |  |  | X3 | 4.082 |
| X4 | 457.260 | 1 | 457.26 | 104.70 | < 0.0001 |  |  | X4 | 4.365 |
| X1X2 | 8.670 | 1 | 8.67 | 1.99 | 0.1792 |  |  | X1X2 | 0.736 |
| X1X3 | 4.582 | 1 | 4.58 | 1.05 | 0.3220 |  |  | X1X3 | -0.535 |
| X1X4 | 0.063 | 1 | 0.06 | 0.01 | 0.9062 |  |  | X1X4 | -0.063 |
| X2X3 | 55.361 | 1 | 55.36 | 12.68 | 0.0028 |  |  | X2X3 | 1.860 |
| X2X4 | 28.724 | 1 | 28.72 | 6.58 | 0.0216 |  |  | X2X4 | -1.340 |
| X3X4 | 0.584 | 1 | 0.58 | 0.13 | 0.7196 |  |  | X3X4 | -0.191 |
| X12 | 32.140 | 1 | 32.14 | 7.36 | 0.016 |  |  | X12 | -1.082 |
| X22 | 43.285 | 1 | 43.29 | 9.91 | 0.0066 |  |  | X22 | -1.256 |
| X32 | 10.291 | 1 | 10.29 | 2.36 | 0.1456 |  |  | X32 | 0.613 |
| X42 | 29.951 | 1 | 29.95 | 6.86 | 0.0194 |  |  | X42 | -1.045 |
| Residual | 65.513 | 15 | 4.37 |  |  |  |  |  |  |
| Lack of Fit | 56.935 | 10 | 5.69 | 3.32 | 0.0988 | Not significant |  |  |  |
| Pure Error | 8.578 | 5 | 1.72 |  |  |  |  |  |  |
| Cor Total | 1975.778 | 29 |  |  |  |  |  |  |  |
| ***Quadratic summary statistics*** | | R^2^ | Adj-R^2^ | Pred-R^2^ | Std. Dev. | C.V. % | | PRESS | Adequate precision |
| Response (R% Dye) | | 0.9668 | 0.9359 | 0.8278 | 2.090 | 2.616 | | 340.3 | 21.06 |

**References**

1. Ghaedi, M.; Hajjati, S.; Mahmudi, Z.; Tyagi, I.; Agarwal, S.; Maity, A. Gupta, V. K. Modeling of competitive ultrasonic assisted removal of the dyes–Methylene blue and Safranin-O using Fe_3_O_4_ nanoparticles. Chem. Eng. J. **2015**, 268, 28–37.
2. Azad, F. N.; Ghaedi, M.; Dashtian, K.; Hajati, S.; Goudarzi, A.; Jamshidi, M. Enhanced simultaneous removal of malachite green and safranin O by ZnO nanorod-loaded activated carbon: modeling, optimization and adsorption isotherms. New J. Chem. **2015**, 39, 7998–8005.
3. M. Ghaedi, A. M. Ghaedi, N. Dehghanian, K. Dashtian, A hybrid model of support vector regression with genetic algorithm for adsorption of malachite green onto multi-walled carbon nanotube forecasting: Central composite design optimization, Phys. Chem. Chem. Phys., **2016**, **18**, 13310-13321.
4. Mazaheri, M. Ghaedi, M. H. Ahmadi Azqhandi and A. Asfaram, Application of machine/statistical learning, artificial intelligence and statistical experimental design for modeling and optimization of methylene blue and Cd (II) removal from binary aqueous solution by natural walnut carbon, **DOI:**10.1039/C6CP08437K.
5. Langmuir, I.; The adsorption of gases on plane surfaces of glass, mica and platinum. J. Am. Chem. Soc. **1918**, 40, 1361–1403.
6. Freundlich, H.; Heller, W. The adsorption of cis-and trans-azobenzene. J. Am. Chem. Soc. **1939**, 61, 2228–2230.
7. Temkin, M. I.; Pyzhev, V.; Kinetics of ammonia synthesis on promoted iron catalysts. Acta Physiochim. URSS. **1940**, 12, 217–222.
8. Dubinin, M. M.; Radushkevich, L. V. Equation of the characteristic curve of activated charcoal. Chem. Zentr. **1947**, 1, 875.
9. Cheng, B., Le, Y., Cai, W., & Yu, J. Synthesis of hierarchical Ni(OH)_2_ and NiO nanosheets and their adsorption kinetics and isotherms to Congo red in water, Journal of Hazardous Materials, **2011,** 185, 889–897.
10. Zeng, S., Duan, S., Tang, R., Li, L., Liu, C., & Sun, D. Magnetically separable Ni_0.6_Fe_2.4_O_4_ nanoparticles as an effective adsorbent for dye removal: synthesis and study on the kinetic and thermodynamic behaviors for dye adsorption, Chemical Engineering Journal, **2014**, 258, 218–228.
